# Biosynthesis and Identification of Clenbuterol Metabolites in Urine and In Vitro Microsome Incubation Samples Using UHPLC‐Q‐Exactive Orbitrap Mass Spectrometry: A Comparison Between Human and Bovine Metabolism

**DOI:** 10.1002/dta.3942

**Published:** 2025-08-26

**Authors:** Anuar Gómez‐Tagle, Claudia Bressan, Rosa Ventura, Alan Álvarez‐Sanchez, Enrique Cardenas‐Yong, Benjamín Velasco‐Bejarano

**Affiliations:** ^1^ Organic Chemistry Section, Chemical Sciences Department, Laboratory of Medicinal Green Chemistry, Faculty of Higher Studies Cuautitlan National Autonomous University of Mexico Cuautitlán Izcalli Mexico; ^2^ Catalonian Anti‐Doping Laboratory Hospital del Mar Institute for Medical Research Barcelona Spain

**Keywords:** bovine microsome assay, clenbuterol metabolites, Orbitrap

## Abstract

Clenbuterol (Clb) is a β2‐agonist drug included in the list of substances prohibited during and out of competition by the World Anti‐Doping Agency (WADA‐AMA). Several adverse analytical findings have been detected by accredited WADA laboratories, but athletes often claim that results are due to dietary contamination. In this contribution, bovine microsomal incubation and the excretion of bovine and human urinary metabolites of Clb were analyzed and compared using liquid chromatography electrospray Q‐Exactive‐Orbitrap mass spectrometry to determine differences in Clb metabolism. Urine samples were processed by solid‐phase extraction prior to electrospray analysis in both the positive and negative ion modes. MS/MS experiments were obtained by parallel monitoring reaction (PRM) triggered by an inclusion ions list. The strategy for metabolite identification involved the search for typical biotransformation based on accurate mass shifts using diagnostic fragment ions from the parent drug. This approach successfully identified eight metabolites, including a novel *N*‐methylated form of Clb, reported here for the first time. Additionally, four metabolites found exclusively in bovine urine offer significant potential for further research aimed at distinguishing unintentional doping.

## Graphical abstract

Clenbuterol metabolism was studied in bovine and human urine using LC‐Q‐Exactive‐Orbitrap MS; eight metabolites were identified in human and bovine samples as well as a novel *N*‐methylated form. Four urinary metabolites specific to cattle were detected and identified. SPE and PRM‐MS/MS enabled metabolite detection and identification, aiding antidoping efforts.

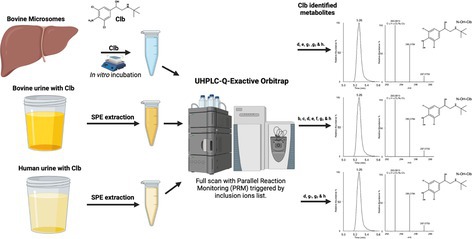

## 1 Introduction

Drug metabolism is a complex process divided into two phases in which a wide variety of enzymatic families participate to catalyze transformation reactions in xenobiotics and eliminate drugs from the body to prevent intoxication [1, 2, 3]. Knowledge of drug metabolism and excretion is of particular interest for antidoping tests, where accurate information on the interval during which the drug, or its metabolites, can be detected is of great importance. This is the case of clenbuterol (Clb), a β_2_‐agonist drug used as a doping agent [4, 5, 6] due to its widely documented ergogenic [7] and lipolytic activity [8], which led it to be included on the World Anti‐Doping Agency's (WADA‐AMA) list of banned substances and prohibited both during and outside competition [9].

In vitro and in vivo studies of drug metabolism have been used routinely in pharmacokinetic analyses of newly synthesized molecules to obtain crucial information on their toxicity [10]. Derived from this, and because hepatic metabolism is the preferred metabolic route for most xenobiotics, liver microsomes are the ones most commonly used in in vitro models for this purpose, since not only a large number of metabolic enzymes—such as cytochrome P450 (CYP) and UDP‐glucuronyltransferases (UGT)—are expressed there, but also many others that participate in these transformations [11, 12, 13].

Among the most important Clb transformations reported for animals in vivo and in vitro experiments are aromatic amine oxidations [14, 15, 16, 17], conjugation with glucuronide [18], and side‐chain oxidations and conjugation with SO_3_ [19]. No useful reports of human metabolites have been made.

Under this premise, and based on prior knowledge that hepatic metabolism differs among species [20, 21, 22, 23], in this contribution, the in vivo and in vitro metabolism of Clb was compared between cattle and humans, a topic on which little information is currently available [24]. Our aim is to develop an analytical detection method using ultra‐high‐performance liquid chromatography coupled with high‐resolution mass spectrometry (UHPLC‐HRMS) in which the retention time of the metabolites generated with bovine hepatic microsomes can be identified to confirm their presence. In this study, urine samples obtained from bovines and healthy human volunteers who consumed Clb were analyzed to correlate the presence of this substance in both sample sets.

Results could provide a way to determine—in future experiments—whether the presence of these metabolites in a human urine sample is due to consumption of contaminated beef that potentially contains them, since they are not metabolically related to humans, according to previous references that suggest that ingesting foods contaminated with Clb [25, 26] can lead to involuntary exposure among athletes [27, 28, 29].

## 2 Experiments

### 2.1 Chemicals and Reagents

Clenbuterol hydrochloride (1‐(4‐amino‐3,5‐dichlorophenyl)‐2‐(*tert*‐butylamine)ethanol) (≥ 95% purity) was purchased from Sigma Chemical Co., St. Louis, USA, and used for microsome assays and as a reference standard. Sucrose, alamethicin ( *T. viride* ), glucose‐6‐phosphate dehydrogenase (G6PDH) ( *S. cerevisiae* ), bovine serum albumin (BSA), phosphoric acid, Coomassie brilliant blue G‐250, and magnesium, sodium, calcium, and potassium chlorides were also purchased from Sigma‐Aldrich. D‐Glucose‐6‐phosphate (G6P) (disodium salt) was acquired from Roche Diagnostics GmbH (Mannheim Germany). Uridine 5′‐diphosphoglucuronic acid trisodium salt (UDPGA) and nicotinamide adenine dinucleotide phosphate (NADP^+^) (hydrate) were obtained from Cayman Chemical Company (Michigan, USA). HPLC/MS grade methanol, chloroform, dichloromethane, and ethanol were acquired from Sigma Aldrich and used without further purification. HPLC‐grade Milli‐Q water was obtained from a Merck Millipore, Milli‐Q Advantage A10 water purification system.

### 2.2 Urine Sample Collection

Urine bovine samples were reused from previous research [30]; samples were stored at −20°C until analysis. Human urine samples were obtained from a healthy male volunteer after administration of 20 μg of Clb every 12 h for 2 days. The clinical protocol was approved by the Local Ethical Committee (CEIC‐IMAS, Institut Municipal d'Assistència Sanitària, Barcelona, Spain). Urine samples were collected before administration and up to 72 h after the first dose and stored at −20°C until analysis.

### 2.3 Bovine Liver Microsome Isolation

Liver tissue samples were collected from bovines (at the Faculty of Higher Studies Cuautitlán, Campus 4, UNAM), immediately after sacrifice at the slaughterhouse. The tissues were weighed (5 g) and cut into small pieces to be washed three times with cold NaCl (0.9%), then suspended in a sucrose solution (0.25 M, 1‐mL/g liver), and homogenized in a high‐speed homogenizer (Lankai model FSH‐2A) at ~12,000 rpm and 4°C until complete tissue disruption was achieved. The microsomal fraction was obtained using the homogenate and centrifuged in a Centurion Ultracentrifuge (Scientific Limited, model 1.K241R [110 V 60 Hz]) at 9700 *g* and 4°C for 10 min. The supernatant was centrifuged again at 18,300 *g* for 25 min at 4°C, following the addition of CaCl_2_ (8 mM) to adjust the solution in order to isolate the microsomal fraction. The supernatant was discarded. The resulting pellet was resuspended in a KCl (0.15 M) solution and centrifuged again at 18,300 *g* and 4°C for 25 min. That supernatant was also discarded to obtain the pellet that contained the microsomal fraction, which was stored at −80°C in a carbonate buffer (pH 7.4, 0.1 M, 20% glycerol) until use. Protein concentration was determined as described by the Bradford assay [31] using bovine serum albumin (BSA) as a standard protein.

### 2.4 In Vitro Microsome Clenbuterol Assays

A dual activity microsomal system in which both CYP and UGTs were active was used as described by Yan et al. [32]. All microsomal incubations were performed in triplicate in 1.5‐mL Eppendorf tubes at 37°C with gentle agitation in a Biomedica Lab thermoblock using bovine liver microsomes. For the first assay, Clb (200 μM) was preincubated for 5 min with bovine microsomes (0.7 mg mL^−1^) in a carbonate buffer (50 mM, pH 7.4, MgCl_2_ 3.5 mM) in the presence of the pore‐forming peptide alamethicin (25 μg mL^−1^). Incubations were initiated by adding an NADPH generation system (NADP^**+**^ 1.3 mM, glucose‐6‐phosphate 3.3 mM, glucose‐6‐phosphate dehydrogenase 0.4 U mL^−1^) for CYP activation and UDPGA (5 mM) for UGT activation to give a total volume of 1 mL.

Second and third control incubations were also performed. In the second, UDPGA and NADPH generation system were not added, while the third was performed without bovine liver microsomes to determine drug stability under those conditions. A final positive control was used to determine the correct functioning of the microsomal system, carried out with testosterone (200 μM) under the conditions mentioned above for the Clb assay. After incubation for 30 min, reactions were stopped by adding ice‐cold acetonitrile (200 μL). The samples were then centrifuged at 18,300 *g* for 25 min at 4°C. The resulting supernatants were transferred directly to micro vials for metabolite profiling using a UHPLC‐HRMS instrument.

### 2.5 Sample Bovine and Human Urine Preparations

Ultracentrifuged (9700 *g*, 10 min) bovine and human urines (5 mL) were passed through a polymeric Waters Sep‐Pak C18 500‐mg cartridge using a Supelco Visiprep 24 (Merck). The cartridges were previously conditioned with 4 mL of methanol and 6 mL of MilliQ water. The analytes were eluted from the cartridge with 4 mL of methanol, and the extracts were evaporated to near dryness with a TurboVap LV (Caliper LifeSciences, Hopkinton, MA) under a nitrogen flow in a water bath at 50°C, then reconstituted with 200 μL of H_2_O:CH_3_CN (90:10). The extract was transferred to a microvial prior to UHPLC‐HRMS analyses.

### 2.6 UHPLC‐Q‐Exactive Orbitrap

UHPLC‐HRMS analyses were performed on a ThermoScientific Vanquish UHPLC system interfaced to a ThermoScientific Q‐Exactive Orbitrap spectrometer. A Waters Acquity UHPLC BEH C18 column (2.1 × 100 mm, 1.7 μm) was used for the chromatographic separations. The mobile phases were A: H_2_O and B: methanol, both with 0.1% v/v formic acid and ammonium formate at 1 mM. The analytes were eluted using a linear gradient from 95% A to 95% B over 20 min at a flow rate of 0.3 mL min^−1^, then 10 μL of each sample were injected. The column chamber temperature was maintained at 45°C throughout the chromatographic acquisitions.

HRMS was conducted using electrospray ionization in both positive and negative ion modes. The capillary voltage was set at 3.0 kV (+/−), and the capillary temperature at 320°C. The temperature of the auxiliary gas heater was 300°C. The sheath gas flow rate and the aux gas flow rate were 50 and 15 arbitrary units, respectively, and the S‐lens RF level was 50. The scan modes were full MS with a resolution of 70,000. The MS^2^ spectra were obtained by parallel reaction monitoring (PRM) triggered by the inclusion ions list in a scan range of *m/z* 65–750. The stepped normalized collision energies were 30, 50, and 70 eV. Data acquisition and processing were carried out with Thermo Xcalibur software (v. 4.1.31.9).

## 3 Results and Discussion

### 3.1 Identification of Clenbuterol and Its Metabolites in Microsome Assays

**FIGURE 1:**
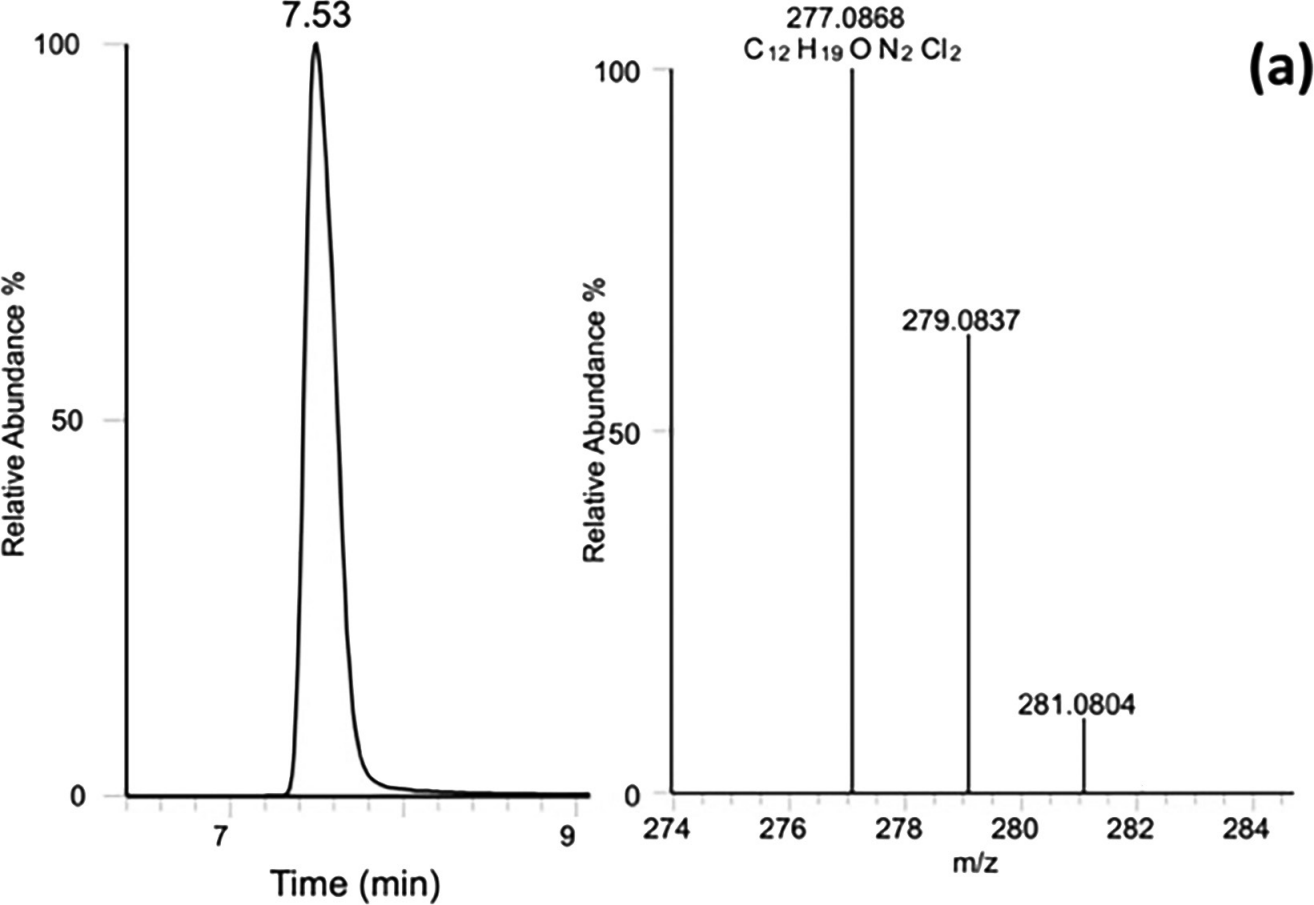
Extracted ion chromatogram and accurate mass spectra isotopic pattern of clenbuterol (a) obtained in full scan mode.

Results of the microsome assays were analyzed to identify Clb and its metabolites and to confirm their presence using UHPLC‐Q‐Exactive Orbitrap accurate mass measurements and the specific retention times for each one. The retention time observed for Clb was 7.5 min with *m*/*z* 277.0868, findings that matched the characteristic ions of the Clb standard (RT: 7.5 min; Figure 1). Minor variations in RT may arise from differences in sample matrices and analysis days.

For purposes of confirmation and the subsequent metabolite search based on diagnostic ions, we performed PRM experiments triggered by the inclusion ions list with stepped normalized collision energies of 30, 50, and 70 eV of clenbuterol. The main fragment ions detected with *m*/*z* 259.0766 (loss of water), *m*/*z* 221.0246 (loss of *tert‐*butyl), *m*/*z* 203.0137 (loss of *tert‐*butyl and water), *m*/*z* 185.9874 (loss of water and *tert*‐butylamine), *m*/*z* 168.0450, and *m*/*z* 132.0684 (consecutive loss of Cl and HCl) ions were also confirmed (Figure S1). Likewise, five Clb metabolites were identified in the bovine microsome samples, as shown in Table 1.

**TABLE 1:** Summary of clenbuterol metabolites in bovine microsome assay via liquid chromatography‐Q‐Exactive Orbitrap HRMS.

Metabolite	ESI mode	Retention time	Experimental *m*/*z* ^a^	Theoretical *m*/*z*	Error (ppm)	Proposed formula
Microsome assays						
Clb	Pos	7.5	277.0875	277.0868	2.038	C_12_H_19_ON_2_Cl_2_
*N*‐OH‐Clb	Pos	5.3	293.0814	293.0818	−1.439	C_12_H_19_O_2_N_2_Cl_2_
NO_2_‐Clb	Pos	10.6	307.0608	307.0610	−0.936	C_12_H_17_O_3_N_2_Cl_2_
Gluc^1^‐Clb	Pos	6.9	453.1183	453.1189	−1.529	C_18_H_27_O_7_N_2_C_l2_
	Neg	6.9	451.1045	451.1044	0.067	C_18_H_25_O_7_N_2_C_l2_
Gluc^2^‐Clb	Pos	7.4	453.1184	453.1189	−1.265	C_18_H_27_O_7_N_2_C_l2_
	Neg	7.4	451.1047	451.1044	0.688	C_18_H_25_O_7_N_2_C_l2_
*N*_ar_‐Met‐Clb	Pos	7.2	291.1030	291.1025	1.459	C_13_H_21_ON_2_Cl_2_

*Note:* Experimental *m*/*z* data and retention times were obtained from PRM fragmentation experiments by inclusion ions list.

^a^ Positive ESI mode, *m*/*z* [M + H], and negative ESI mode, *m*/*z* [M − H].

**FIGURE 2:**
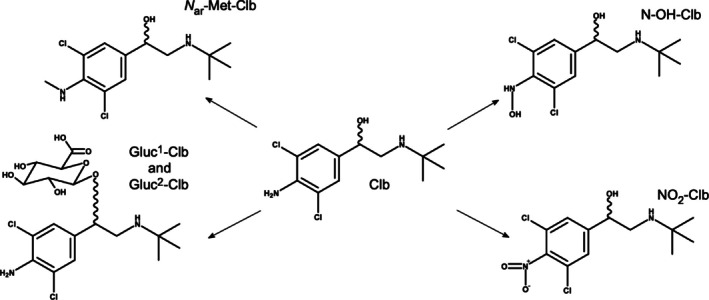
Clb metabolites identified by microsomal assay, proposed by HRMS data.

**FIGURE 3:**
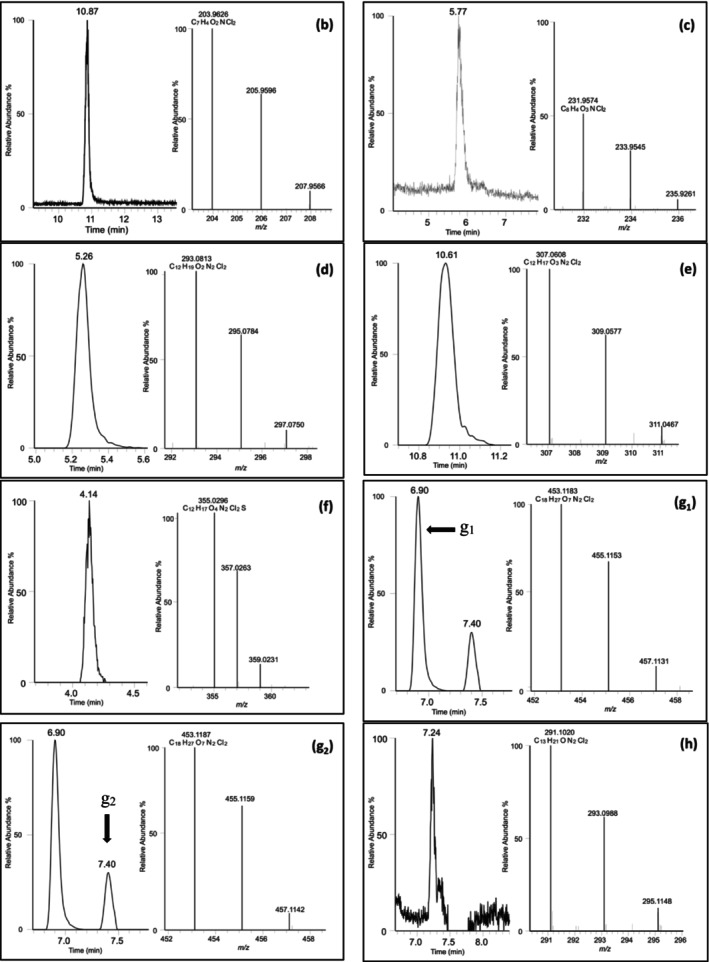
Extracted ion chromatograms and accurate mass spectra isotopic patterns of clenbuterol metabolites identified in full scan mode. (b) ADBA; (c) ADOA; (d) *N*‐OH‐Clb; (e) NO_2_‐Clb; (f) SO_3_‐Clb; (g_1_) Gluc^1^‐Clb; (g_2_) Gluc^2^‐Clb; and (h) *N*_ar_‐Met‐Clb.

These Clb metabolites were generated by a bio‐enzymatic methodology. Their structures were proposed according to the data obtained by Q‐Orbitrap high‐resolution mass spectrometry. They include oxidation of the Clb aniline moiety (*N*‐OH‐Clb and NO_2_‐Clb), two phase‐2 metabolites that resulted from conjugation with glucuronic acid (Gluc^1^‐Clb and Gluc^2^‐Clb), and methylation of the amine group in the aromatic ring (*N*_ar_‐Met‐Clb). Figure 2 shows the structures proposed. Extracted ion chromatograms and the accurate mass spectra isotopic pattern of the clenbuterol metabolites identified are depicted in Figure 3.

*N*‐hydroxylation of the aniline moiety of Clb produced the metabolite *N*‐OH‐Clb (hydroxylamine clenbuterol) observed in full scan mode (Figure 3) at a retention time of 5.3 min, *m*/*z* 293.0813. The ion isolation and PRM fragmentation (positive mode) experiments for this metabolite showed fragments at *m*/*z* 275.0713 (loss of water), *m*/*z* 219.0088 (loss of water and *tert*‐butyl moiety), and *m*/*z* 202.0060 (odd electron fragmentation of the remaining hydroxyl group). Both ions were consistent with the ESI‐TOF CID fragmentation data reported by Domínguez‐Romero et al. [33]. The ions *m*/*z* 167.0372 and *m*/*z* 132.0684 (consecutive loss of Cl) were also detected (Figure S2).

The oxidation of *N*‐OH‐Clb produced NO_2_‐Clb, as proposed by Zalko et al. [19, 34] observed in full scan mode (Figure 3) at a retention time of 10.6 min, *m*/*z* 307.0608. Ion isolation and PRM fragmentation experiments for this metabolite showed fragments at *m*/*z* 250.9986 (loss of *tert*‐butyl), *m*/*z* 232.9880 (loss of *tert*‐butyl and H_2_O), and *m*/*z* 186.9951 (loss of *tert*‐butyl, H_2_O and NO_2_). The ions *m*/*z* 152.0263 and *m*/*z* 117.0576 (consecutive loss of Cl ions) were also detected (Figure S3). Regarding the conjugate phase II metabolites, two plausible structures were identified, as reported in the literature [35], as *O*‐glucuronides conjugates (Gluc^1^‐Clb and Gluc^2^‐Clb), with one new form proposed as *N*‐methyl metabolite (*N*_ar_‐Met‐Clb). For Clb glucuronidation, the ion chromatogram of theoretical *m*/*z* 453.1189, as reported by Alonen et al. [18] showed two peaks at distinct retention times: 6.9 (Gluc^1^‐Clb) and 7.4 min (Gluc^2^‐Clb), as shown in Figure 3 (g_1_, g_2_). For the ESI positive mode, the full scan of Gluc^1^‐Clb showed *m*/*z* 453.1183. Ion isolation and PRM fragmentation experiments for this metabolite showed fragments at *m*/*z* 259.0764 (loss of glucuronic acid), *m*/*z* 203.0137 (loss of glucuronic acid and tert‐butyl moiety), *m*/*z* 185.9872 (loss of glucuronic acid and tert‐butylamine moiety and NH_3_), *m*/*z* 168.0449, *m*/*z* 132.0683 (consecutive loss of Cl and HCl) (Figure S4). Similar spectra were detected for Gluc^2^‐Clb, which showed *m*/*z* 453.1187 in full scan mode. Ion isolation and PRM fragmentation showed *m*/*z* 259.0765, *m*/*z* 203.0139, *m*/*z* 185.9874, *m*/*z* 168.0450, and *m*/*z* 132.0684.

For the ESI negative mode, the full scan of Gluc^2^‐Clb showed *m*/*z* 451.1047. PRM fragmentation experiments showed *m*/*z* 257.0619 (loss of glucuronic acid moiety and H_2_). The ions *m*/*z* 193.0355, *m*/*z* 175.0250, *m*/*z* 113.0246, and *m*/*z* 85.0296 were also detected (Figure S5). These last fragments are typical of glucuronide moiety fragmentation, as described by Stachulski et al. [36]. Similar results were obtained for Gluc^2^‐Clb, which showed *m*/*z* 451.1050, *m*/*z* 257.0619, *m*/*z* 193.0355, *m*/*z* 175.0249, *m*/*z* 113.0246, and *m*/*z* 85.0296. *N*‐methylation of clenbuterol has not been reported in the literature on bovine metabolic assays or in its fragmented form. For the ESI positive mode, the full scan of *N*_ar_‐Met‐Clb appeared at a retention time of 7.2 min and *m/z* 291.1020 for the molecular ion. Ion isolation and PRM fragmentation experiments showed *m/z* 273.0922 (loss of water), indicating that methylation could not occur at the alcohol moiety of Clb. The *m/z* 235.0401 (loss of *tert*‐butyl) and *m/z* 217.0295 (loss of *tert*‐butyl and water) ions were also detected. To distinguish the *N*‐methylation site, the diagnostic ion *m/z* 188.0030 (loss of *tert*‐butyl, water, and CH_2_NH) helped differentiate it by the proposed stable tropylium ring formation. The *m/z* 182.0607 and *m/z* 146.0840 ions were also observed due to consecutive Cl and HCl loss (Figure S6).

### 3.2 Identification of Clenbuterol Metabolites in Bovine Urine

Seven Clb metabolites were identified in the bovine urine samples. Table 2 displays the retention times and experimental *m*/*z* values determined. For confirmation and subsequent metabolite identification based on diagnostic ions, parallel reaction monitoring (PRM) was carried out using an inclusion ions list. This analysis applied stepped normalized collision energies of 30, 50, and 70 eV for each Clb metabolite.

**TABLE 2:** Summary of clenbuterol metabolites in bovine urine detected using LC‐Q‐Exactive Orbitrap HRMS.

Metabolite	ESI mode	Retention time	Experimental *m*/*z* ^b^	Theoretical *m*/*z*	Error (ppm)	Proposed formula
Bovine urine						
Clb	Pos	7.6	277.0869	277.0868	−0.045	C_12_H_19_ON_2_Cl_2_
ADBA	Neg	10.7	203.9627	203.9624	1.289	C_7_H_4_O_2_NCl_2_
ADOA	Neg	5.9	231.9578	231.9568	1.76	C_8_H_4_O_3_NCl_2_
*N*‐OH‐Clb	Pos	5.2	293.0818	293.0818	−0.068	C_12_H_19_O_2_N_2_Cl_2_
NO_2_‐Clb	Pos	10.6	[M + H] not detected^a^	307.0610	NC	C_12_H_17_O_3_N_2_Cl_2_
SO_3_‐Clb	Neg	4.1	355.0295	355.0291	2.450	C_12_H_17_O_4_N_2_SCl_2_
Gluc2‐Clb	Pos	7.4	453.1192	453.1189	0.413	C_18_H_27_O_7_N_2_C_l2_
	Neg	7.4	451.1052	451.1044	1.619	C_18_H_25_O_7_N_2_C_l2_
*N*_ar_‐Met‐Clb	Pos	7.2	291.1024	291.1025	−0.327	C_13_H_21_ON_2_Cl_2_

*Note:* Experimental *m*/*z* data and retention times were obtained from PRM fragmentation experiments by inclusion ions list.

Abbreviation: NC: not calculated.

^a^ Detected by PRM fragmentation pattern and retention time.

^b^ Positive ESI mode, *m*/*z* [M + H], and negative ESI mode, *m*/*z* [M − H].

Unaltered Clb was detected at a retention time of 7.6 min in extracted bovine urine, with experimental *m*/*z* 277.0869. PRM fragmentation experiments showed fragmentation similar to that described above for Clb in Section 3.1. As indicated previously, the minor variations in RT may arise from differences in sample matrices and analysis days.

Using the in vitro microsomal metabolites as a reference, 4 metabolites were detected in bovine urine: *N*‐OH‐Clb, NO_2_‐Clb, Gluc^2^‐Clb, and *N*_ar_‐Met‐Clb, with retention times of 5.2, 10.6, 7.4, and 7.2 min, respectively. PRM fragmentation experiments showed similar fragmentation ions to those described above in Section 3.1 for these metabolites. Neither the Gluc^1^‐Clb molecular ion nor fragments of it were detected in the bovine urine samples.

During the analysis of the bovine samples in full scan, three more metabolites were observed in addition to the compounds just described. They correspond to the oxidative cleavage of the side chain (ADBA, 4‐amino‐3,5‐dichloro benzoic acid and ADOA, 2‐(4‐amino‐3,5‐dichlorophenyl)‐2‐oxoacetic acid) with retention times of 10.9 min, *m*/*z* 203.9626, for ADBA and 5.8 min, *m*/*z* 231.9574, for ADOA, and an *N*_*ar*_‐sulfation (SO_3_‐Clb) of the aniline clenbuterol moiety with a retention time of 4.1 min, *m*/*z* 355.0296. Figure 3 shows the extracted ion chromatograms and accurate mass spectra isotopic patterns of the Clb metabolites identified.

The metabolites ADBA and ADOA were identified as forms generated by the oxidative cleavage of the side chain of clenbuterol, as described previously in the literature [33, 34], though they have never been identified or fragmented. PRM fragmentation experiments (negative mode) for ADBA showed characteristic fragments at *m*/*z* 159.9729 (loss of CO_2_) and *m/z* 123.9961 (loss of CO_2_ and HCl) (Figure S7). The fragments for ADOA showed *m*/*z* 203.9628 (loss of CO) via an intramolecular rearrangement. The ions *m*/*z* 159.9729 (loss of CO_2_) and *m*/*z* 123.9962 (loss of CO_2_ and HCl) were also detected (Figure S8).

*N*_*ar*_‐sulfation (SO_3_‐Clb) was found in the bovine urine with *m*/*z* 355.0295 in full scan mode (Figure 3). PRM fragmentation experiments (negative mode) for this metabolite showed fragments at *m*/*z* 319.0527 (loss of HCl), *m*/*z* 275.0723 (loss of SO_3_), *m*/*z* 257.0618 (loss of SO_3_ and water), and *m*/*z* 239.9296 (loss of clenbuterol *N*_ar_‐sulfate lateral chain C_6_H_13_NO) (Figure S9).

### 3.3 Identification of Clenbuterol Metabolites in Human Urine

In general, unchanged Clb and four Clb metabolites were identified in human urine samples. Table 3 presents the retention times and *m*/*z* values. For confirmation purposes and the subsequent metabolite search based on diagnostic ions, PRM triggered by inclusion ions list with stepped normalized collision energies of 30, 50, and 70 eV of clenbuterol was performed for each metabolite found.

**TABLE 3:** Summary of clenbuterol metabolites detected in human urine by liquid chromatography‐Q‐Exactive Orbitrap HRMS.

Metabolite	ESI mode	Retention time	Experimental *m*/z^b^	Theoretical *m*/*z*	Error (ppm)	Proposed formula
Human urine						
Clb	Pos	7.6	277.0869	277.0868	−0.055	C_12_H_19_ON_2_Cl_2_
*N*‐OH‐Clb	Pos	5.3	293.0816	293.0818	−0.613	C_12_H_19_O_2_N_2_Cl_2_
Gluc^1^‐Clb	Pos	6.9	[M + H] not detected^a^	453.1189	NC	C_18_H_27_O_7_N_2_C_l2_
Gluc^2^‐Clb	Pos	7.4	[M + H] not detected^a^	453.1189	NC	C_18_H_27_O_7_N_2_C_l2_
*N*_ar_‐Met‐Clb	Pos	7.2	291.1024	291.1025	−0.636	C_13_H_21_ON_2_Cl_2_

*Note:* Experimental *m*/*z* data and retention times were obtained from PRM fragmentation experiments by inclusion ions list.

Abbreviation: NC: not calculated.

^a^ Detected by PRM fragmentation pattern and retention time.

^b^ Positive ESI mode, *m*/*z* [M + H].

Using the in vitro microsomal metabolites and the results from our analysis of the bovine urine samples as references, four metabolites were detected in human urine: *N*‐OH‐Clb, Gluc^1^‐Clb, Gluc^2^‐Clb, and Met‐Clb. The retention times observed for these metabolites are consistent with those found previously in microsomal assays and bovine urine samples tested. PRM fragmentation experiments showed similar fragmentation ions to those described above in Section 3.1 for these metabolites. Gluc^1^‐Clb and Gluc^2^‐Clb were identified in PRM experiments since no [M + H] ions were detected, likely due to very low concentrations.

### 3.4 Comparison of Bovine and Human Metabolites

**FIGURE 4:**
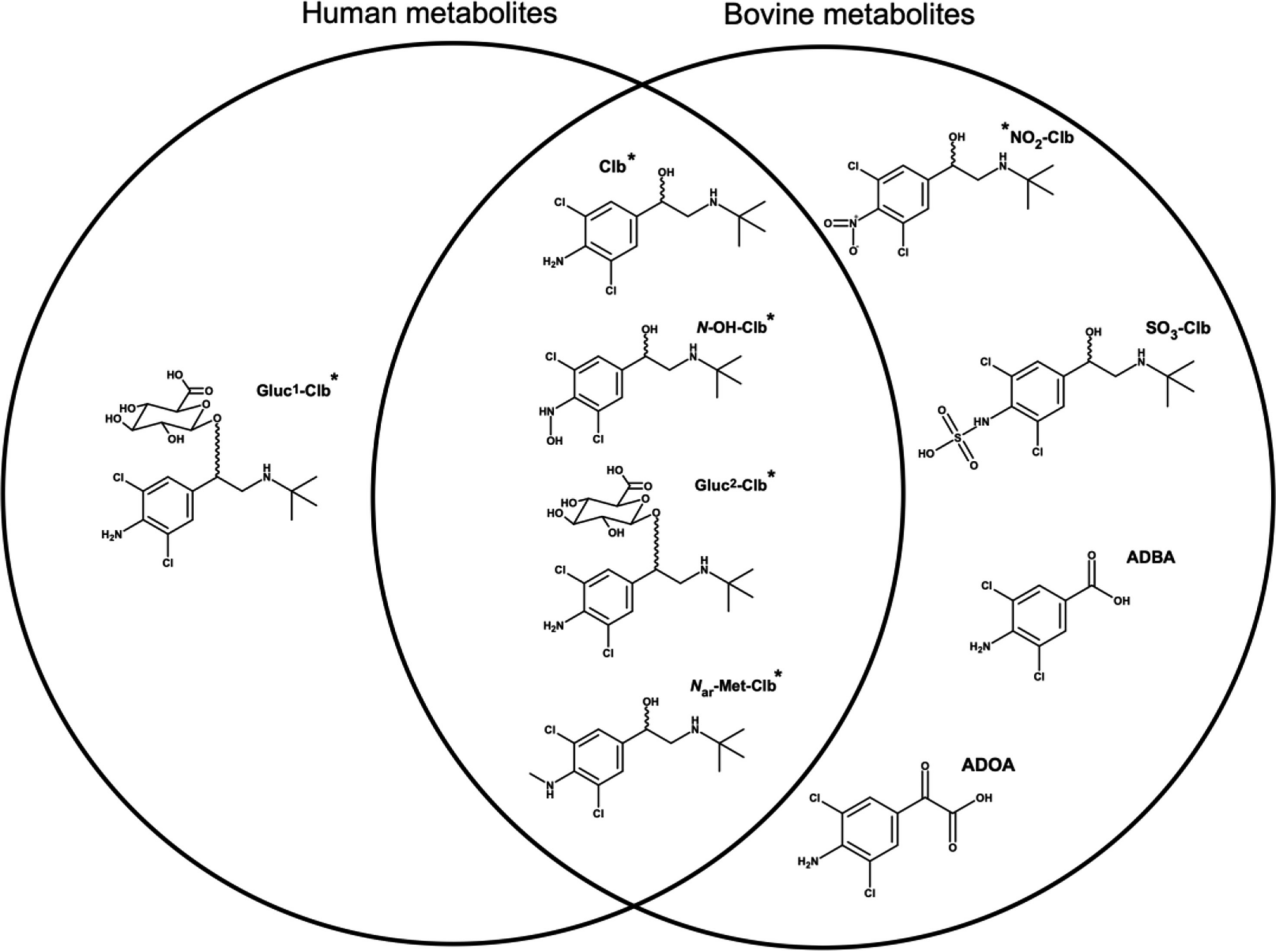
Venn diagram of Clb metabolites identified in human and bovine urine samples, proposed by HRMS data. *Metabolites detected in the in vitro microsome assay.

Overall, eight metabolites were identified in microsomal assays and the urine samples analyzed, but only four could be confirmed in the human urine samples (Figure 4). Extracted ion chromatograms and the full scan mass spectra of each metabolite detected in the microsomal assays or the urine samples are shown in Figure 3, including the two phase I metabolism reactions, that is, (1) oxidative cleavage of the side chain (ADBA, ADOA) and (2) oxidation of the clenbuterol aniline moiety (*N*‐OH‐Clb and NO_2_‐Clb). Three phase 2 reactions were detected: (1) conjugation with sulfate (SO_3_‐Clb); (2) conjugation with glucuronic acid (Gluc^1^‐Clb and Gluc^2^‐Clb); and (3) methylation of the amine group in the aromatic ring (Met‐Clb). Figure 4 shows the proposed structures.

This analysis clearly confirms that the metabolism of Clb differs markedly between humans and bovines. Table 4 shows the main differences observed in the urinary metabolites excreted. Metabolism in the samples of human urine showed complete oxidation of Clb's aromatic amine to nitro (NO_2_‐Clb), sulfation of this amine (SO_3_‐Clb), and the oxidative cleavage of the side chain that produced two distinct metabolites (ADBA and ADOA).

**TABLE 4:** Summary and comparison of the clenbuterol metabolites present in bovine and human urine samples by UHPLC‐Q‐Exactive Orbitrap spectrometry.

Detection and comparison of clenbuterol metabolites in human and bovine urine samples.				
Metabolite	ESI	Precursor (*m*/*z*)^a^	Human	Bovine
Clb	Pos	277.0868	Detected	Detected
N‐OH‐Clb	Pos	293.0818	Detected	Detected
NO_2_‐Clb	Pos	307.0610	Not detected	Detected
Gluc^1^‐Clb	Pos	453.1189	Detected	Not detected
Gluc^2^‐Clb	Pos	453.1189	Detected	Detected
*N*_ar_‐Met‐Clb	Pos	291.1025	Detected	Detected
ADBA	Neg	203.9624	Not detected	Detected
ADOA	Neg	231.9568	Not detected	Detected
SO_3_‐Clb	Neg	451.1044	Not detected	Detected

^a^ Positive ESI mode *m*/*z* [M + H], negative ESI mode *m*/*z* [M − H].

The methods developed for this study should be suitable for future research on Clb metabolism, including comparisons of species. Moreover, it may be possible to adapt them to aid in current problems of adverse analytical results in antidoping tests due to accidental clenbuterol ingestion from contaminated meat. However, additional experiments are required to further elucidate this problem.

## 4 Conclusion

The excretion and comparison of bovine and human urinary metabolites of Clb was studied using UHPLC‐Q‐Exactive Orbitrap mass spectrometry. The strategy applied to identify metabolites was based on UHPLC‐Q‐Exactive Orbitrap analyses using PRM experiments with precursor ion isolation in positive and negative modes to search for common diagnostic fragment ions from the parent drug, coupled with a search for typical biotransformations and their corresponding accurate mass shift. The approach was applied satisfactorily and succeeded in identifying eight metabolites, three of which had not been detected previously, either in urine or as fragments. The new metabolites found in bovine urine included N‐methylation of the aniline moiety of Clb and the presence of ADBA and ADOA.

Our comparison of human and bovine Clb urinary metabolites obtained three common ones (*N*‐OH‐Clb, Gluc^2^‐Clb, *N*_ar_‐Met‐Clb) and five non‐shared forms (ADBA, ADOA, NO_2_‐Clb, Gluc^1^‐Clb, SO_3_‐Clb). These differences may be of interest for doping control analyses in athletes who have had adverse analytical results for clenbuterol allegedly due to the ingestion of contaminated meat. However, further studies are needed to test this affirmation.

## Conflicts of Interest

The authors declare no conflicts of interest.

## Supporting information


**Figure S1:** PRM spectra and fragmentation pattern proposed for CLB. ESI+.
**Figure S2:** PRM spectra and fragmentation pattern proposed for N‐OH‐Clb. ESI+.
**Figure S3:** PRM spectra and fragmentation pattern proposed for NO2‐Clb. ESI+.
**Figure S4:** PRM spectra and fragmentation pattern proposed for Gluc1‐2‐Clb. ESI+.
**Figure S5:** PRM spectra and fragmentation pattern proposed for Gluc1‐2‐Clb. ESI−.
**Figure S6:** PRM spectra and fragmentation pattern proposed for Nar‐Met‐Clb. ESI+.
**Figure S7:** PRM spectra and fragmentation pattern proposed for ADBA. ESI−.
**Figure S8:** PRM spectra and fragmentation pattern proposed for ADOA. ESI−.
**Figure S9:** PRM spectra and fragmentation pattern proposed for SO3‐Clb. ESI–.

## Data Availability

The data that support the findings of this study are available on request from the corresponding author. The data are not publicly available due to privacy or ethical restrictions.
