# Supplementary material for: Biosynthesis and Identification of Clenbuterol Metabolites in Urine and In Vitro Microsome Incubation Samples Using UHPLC‐Q‐Exactive Orbitrap Mass Spectrometry: A Comparison Between Human and Bovine Metabolism
Source: Drug Test Anal. 2025 Aug 26;17(12):2314–22. doi: 10.1002/dta.3942 (PMC12689244; doi:10.1002/dta.3942)

SUPPLEMENTARY INFORMATION

CONTENT

Supplementary figure 1. PRM spectra and fragmentaton pattern proposed for CLB. ESI+ 2

Supplementary figure 2. PRM spectra and fragmentaton pattern proposed for N-OH-Clb. ESI+ 3

Supplementary figure 3. PRM spectra and fragmentaton pattern proposed for NO_2-Clb. ESI+ 4

Supplementary figure 4. PRM spectra and fragmentaton pattern proposed for Gluc1-2-Clb. ESI+ 5

Suplementary figure 5. PRM spectra and fragmentaton pattern proposed for Gluc1-2-Clb. ESI - 6

Supplementary figure 6. PRM spectra and fragmentaton pattern proposed for N_{ar}-Met-Clb. ESI+ 7

Supplementary figure 7. PRM spectra and fragmentaton pattern proposed for ADBA. ESI - 8

Supplementary figure 8. PRM spectra and fragmentaton pattern proposed for ADOA. ESI - 9

Supplementary figure 9. PRM spectra and fragmentaton pattern proposed for SO3-Clb. ESI - 10

Supplementary Figure 1. PRM spectra and fragmentation pattern proposed for CLB. ESI (+)

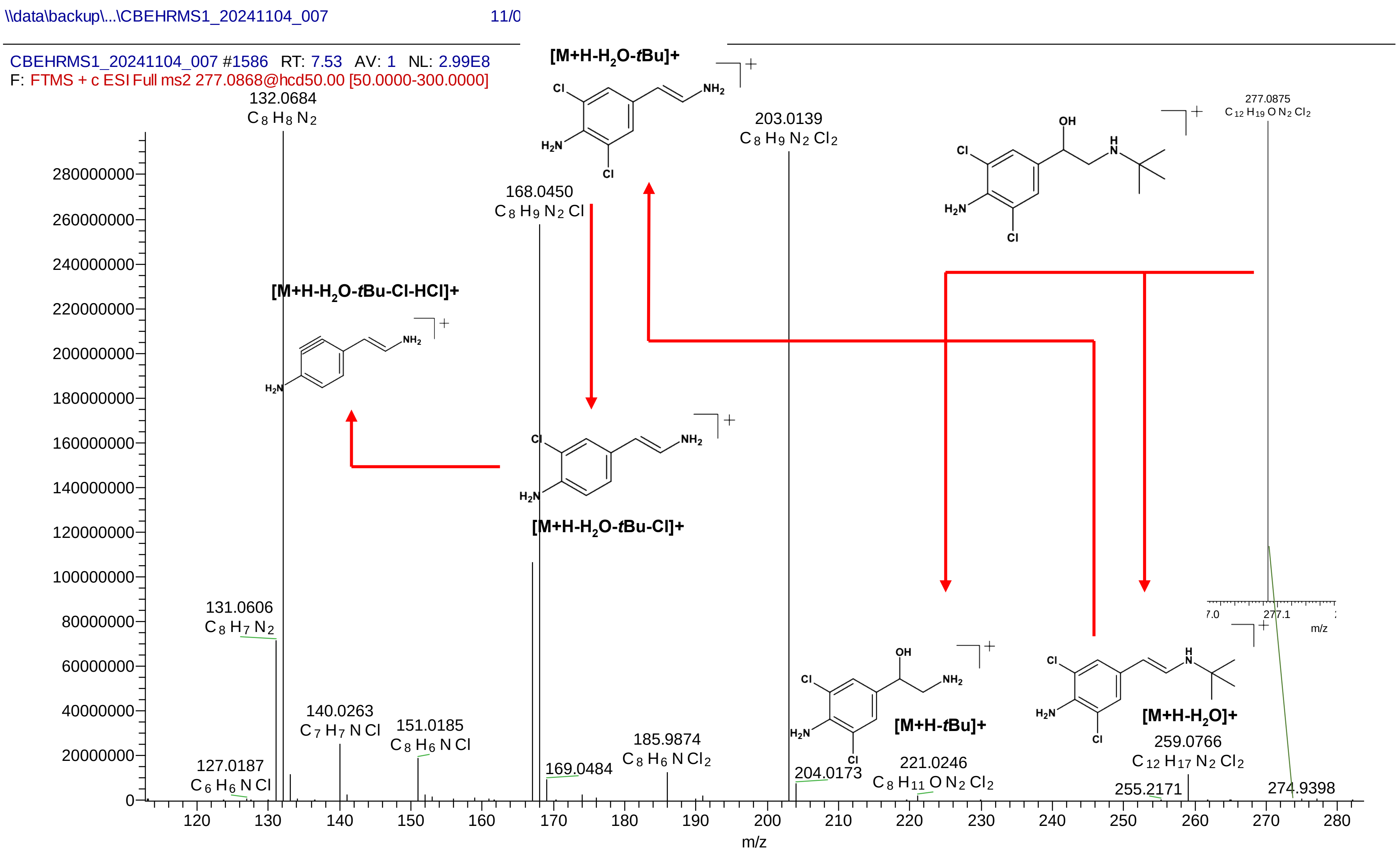

Supplementary Figure 2. PRM spectra and fragmentation pattern proposed for N-OH-Clb. ESI (+)

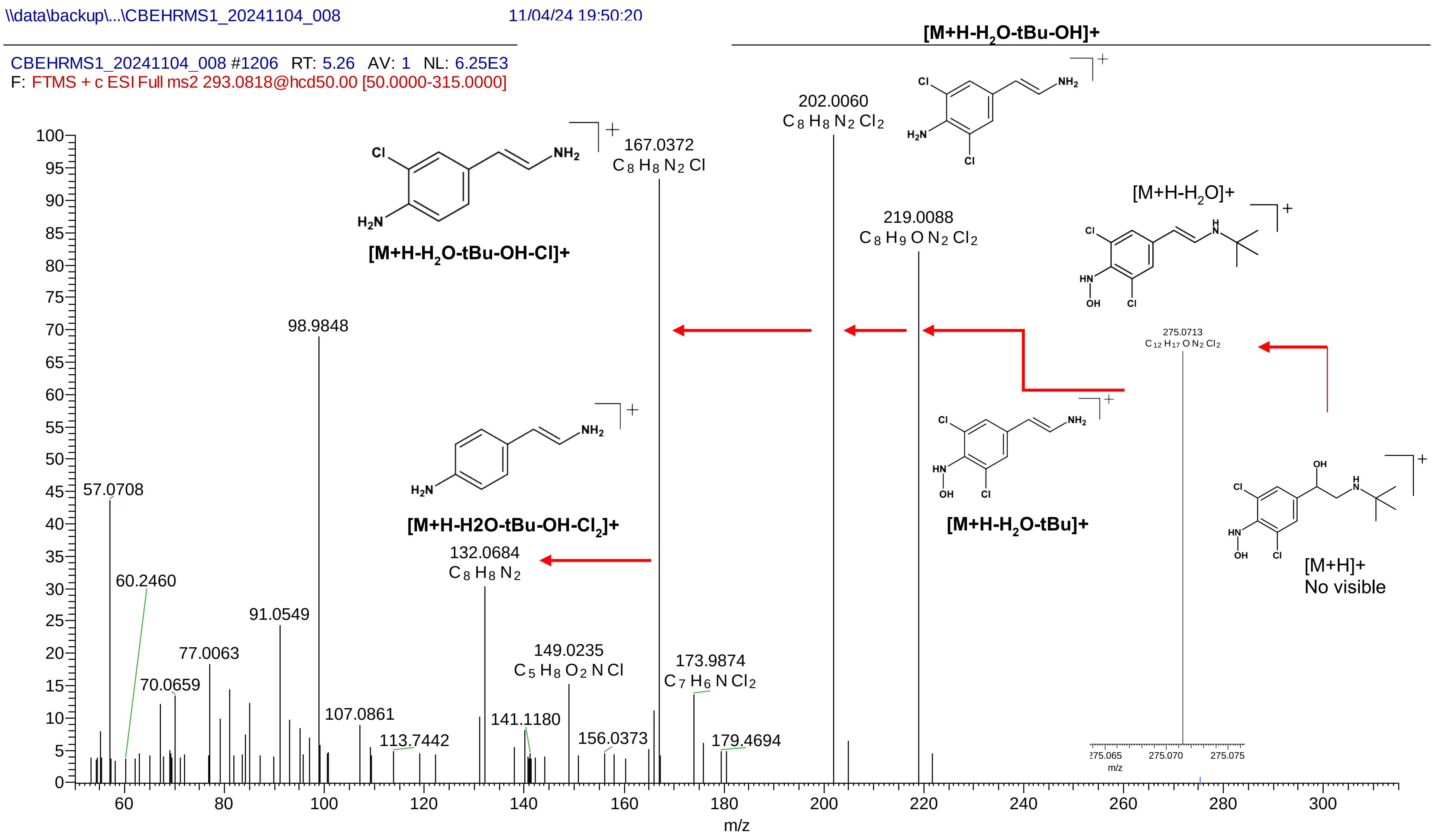

Supplementary Figure 3. PRM spectra and fragmentation pattern proposed for NO_2-Clb. ESI (+)

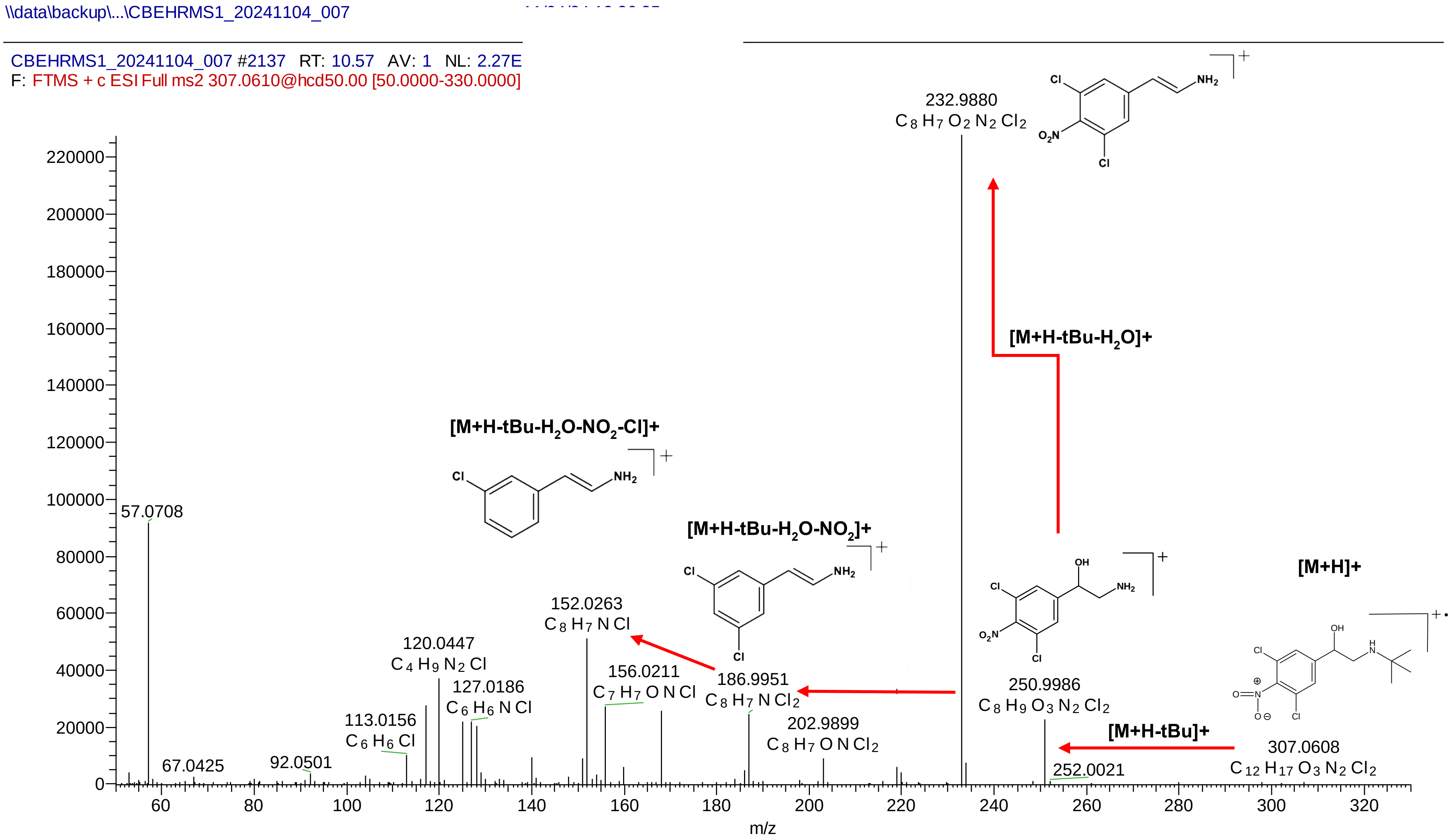

Supplementary Figure 4. PRM spectra and fragmentation pattern proposed for Gluc1-2-Clb. ESI (+)

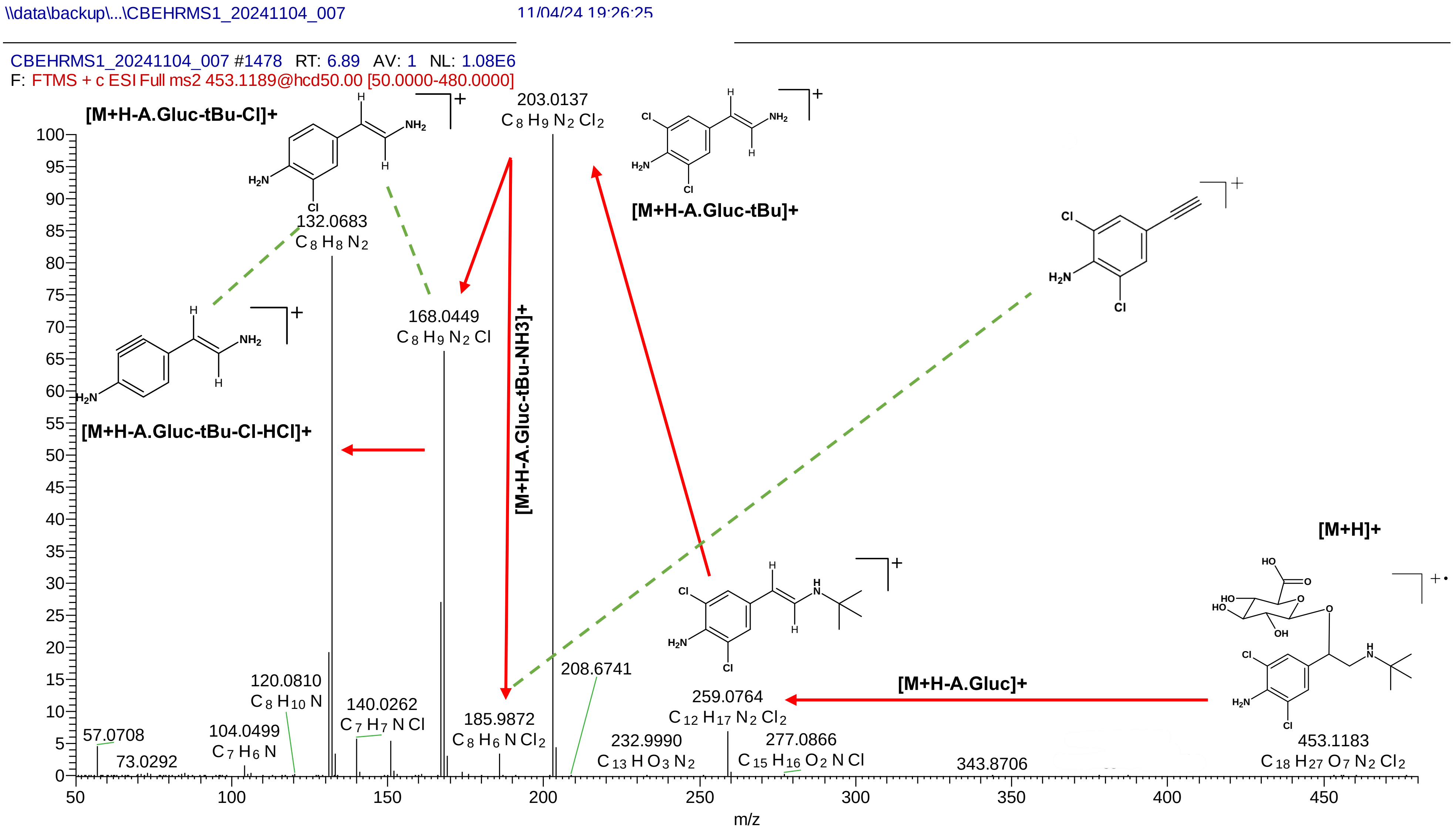

Supplementary Figure 5. PRM spectra and fragmentation pattern proposed for Gluc1-2-Clb. ESI (-)

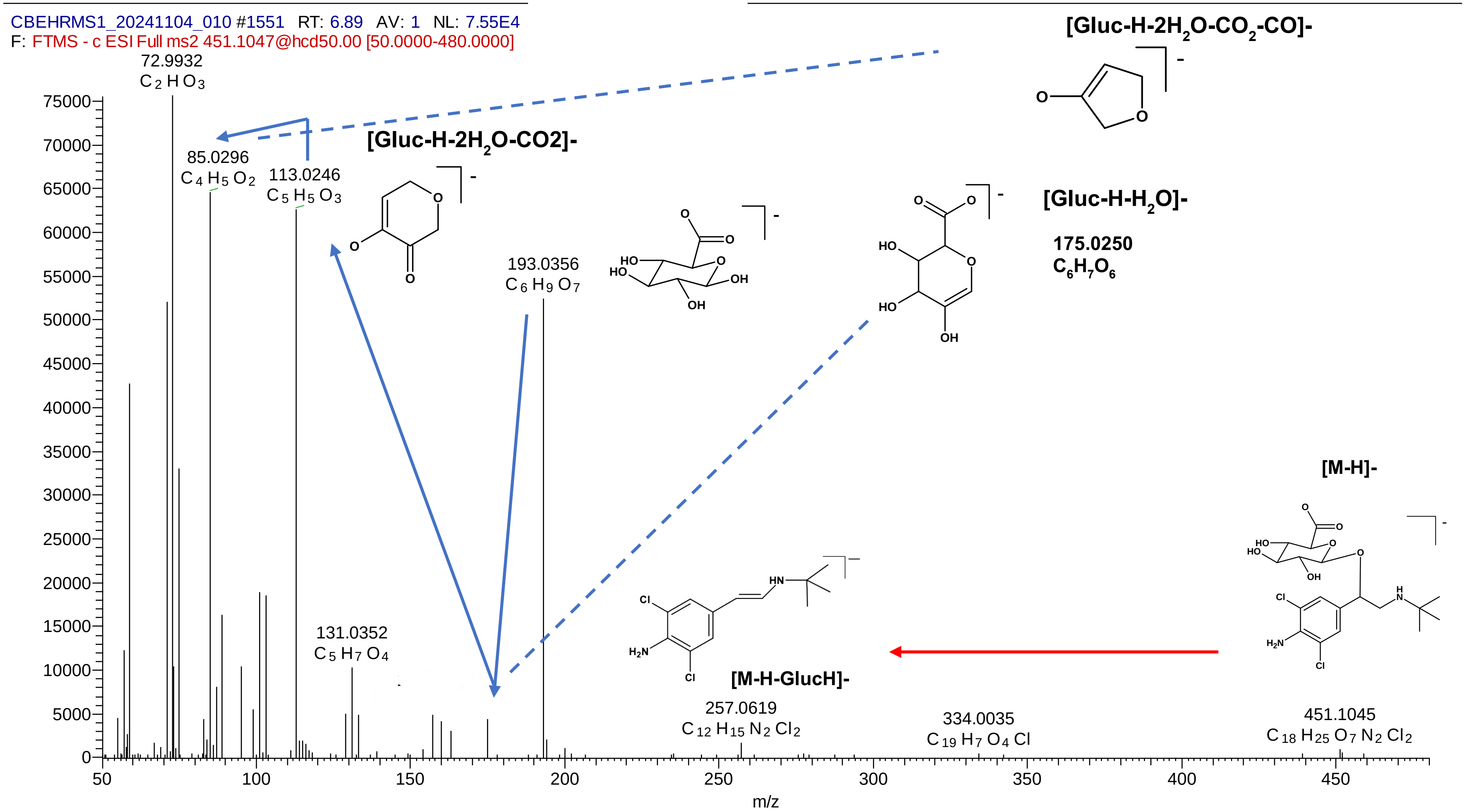

Supplementary Figure 6. PRM spectra and fragmentation pattern proposed for N_{ar}-Met-Clb. ESI (+)

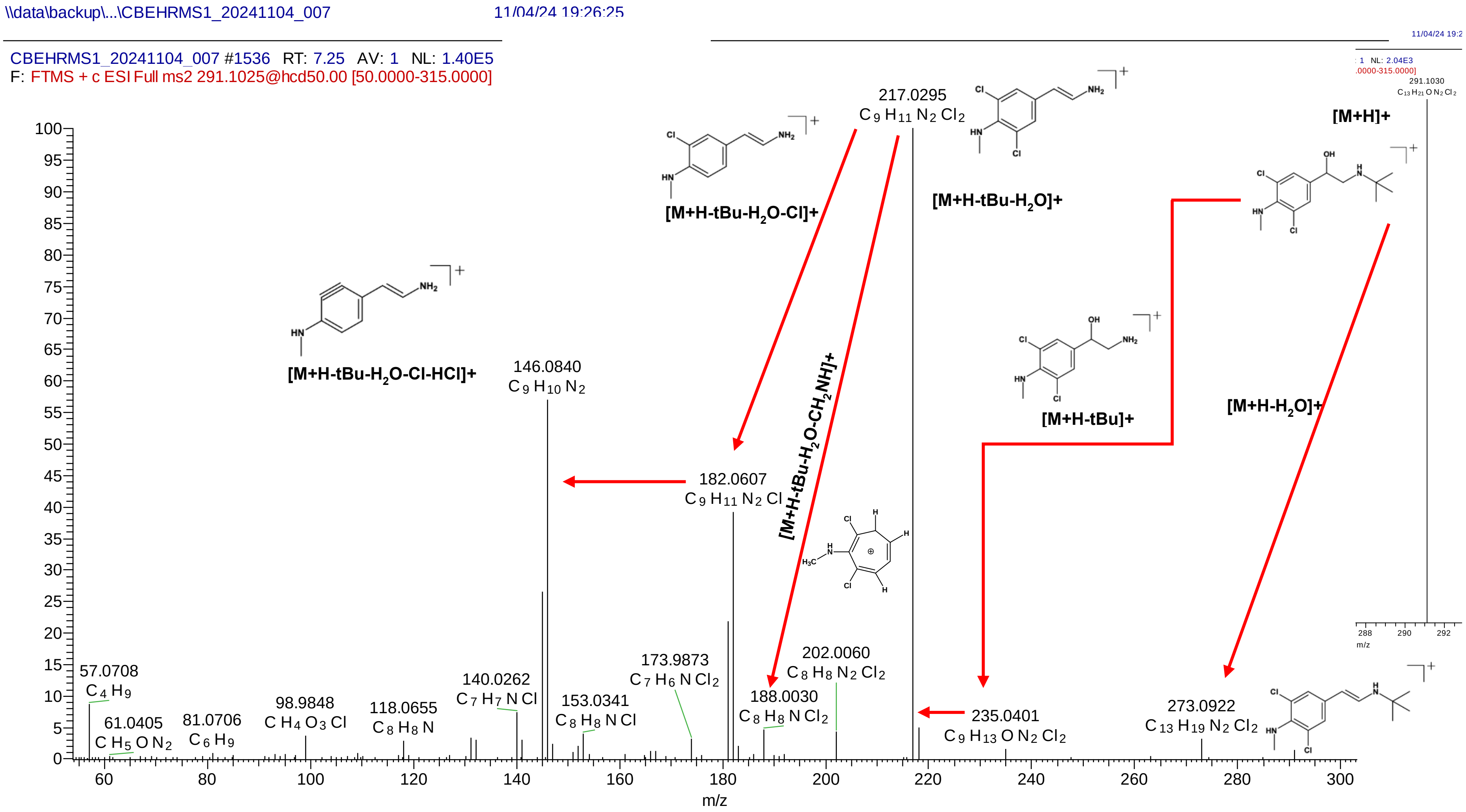

Supplementary Figure 7. PRM spectra and fragmentation pattern proposed for ADBA. ESI (-)

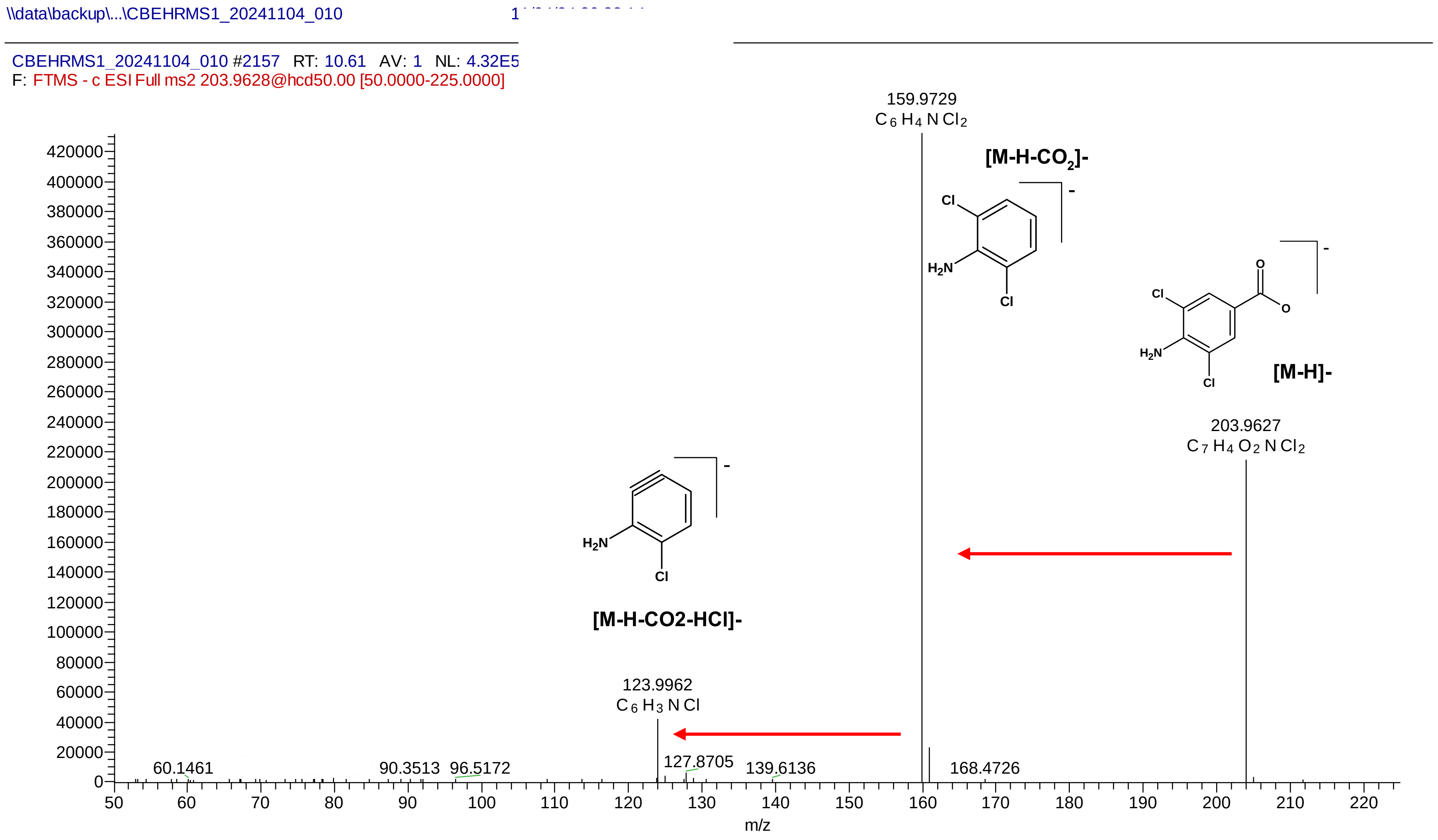

Supplementary Figure 8. PRM spectra and fragmentation pattern proposed for ADOA. ESI (-)

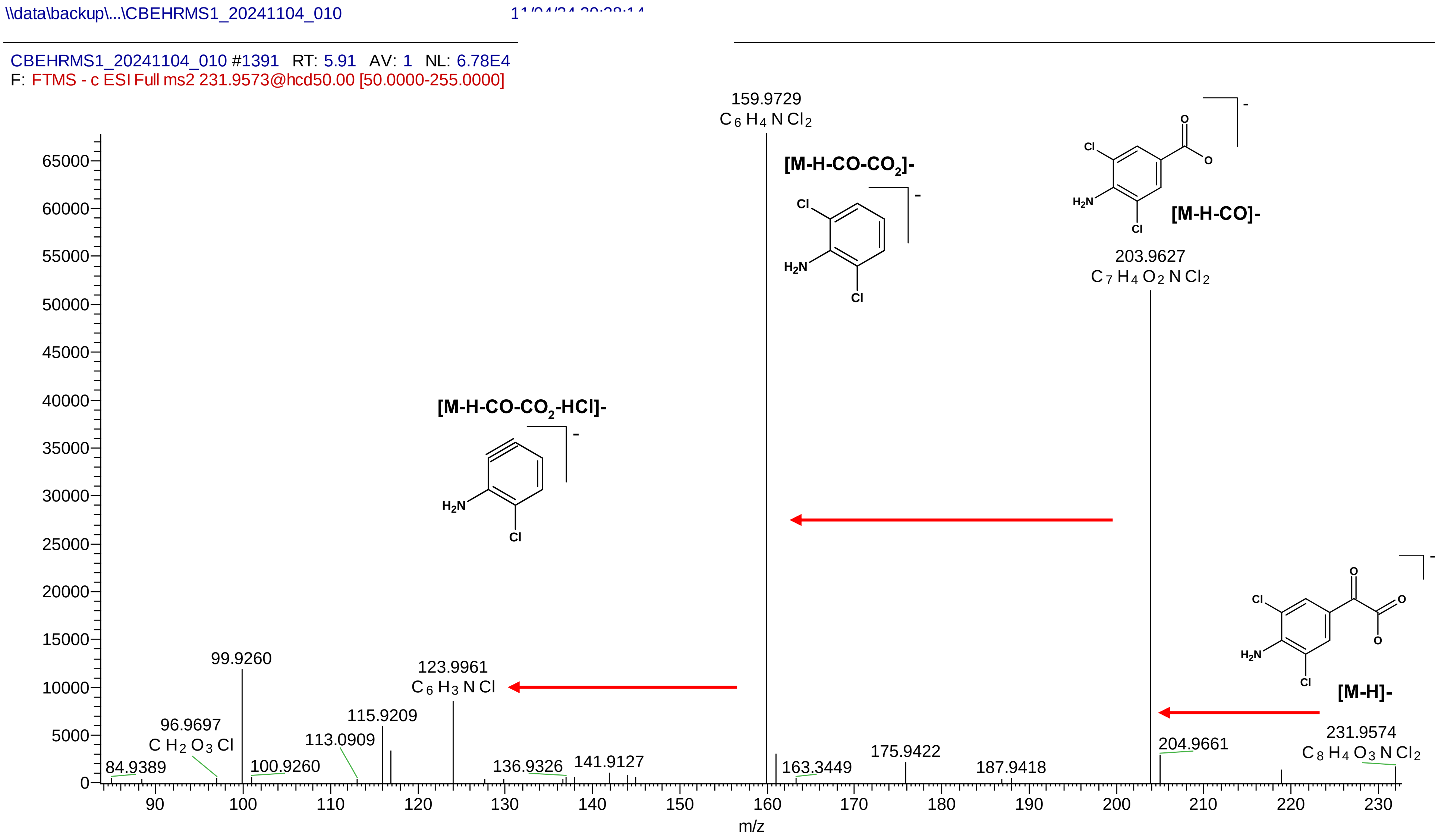

Supplementary Figure 9. PRM spectra and fragmentation pattern proposed for SO_3-Clb. ESI (-)

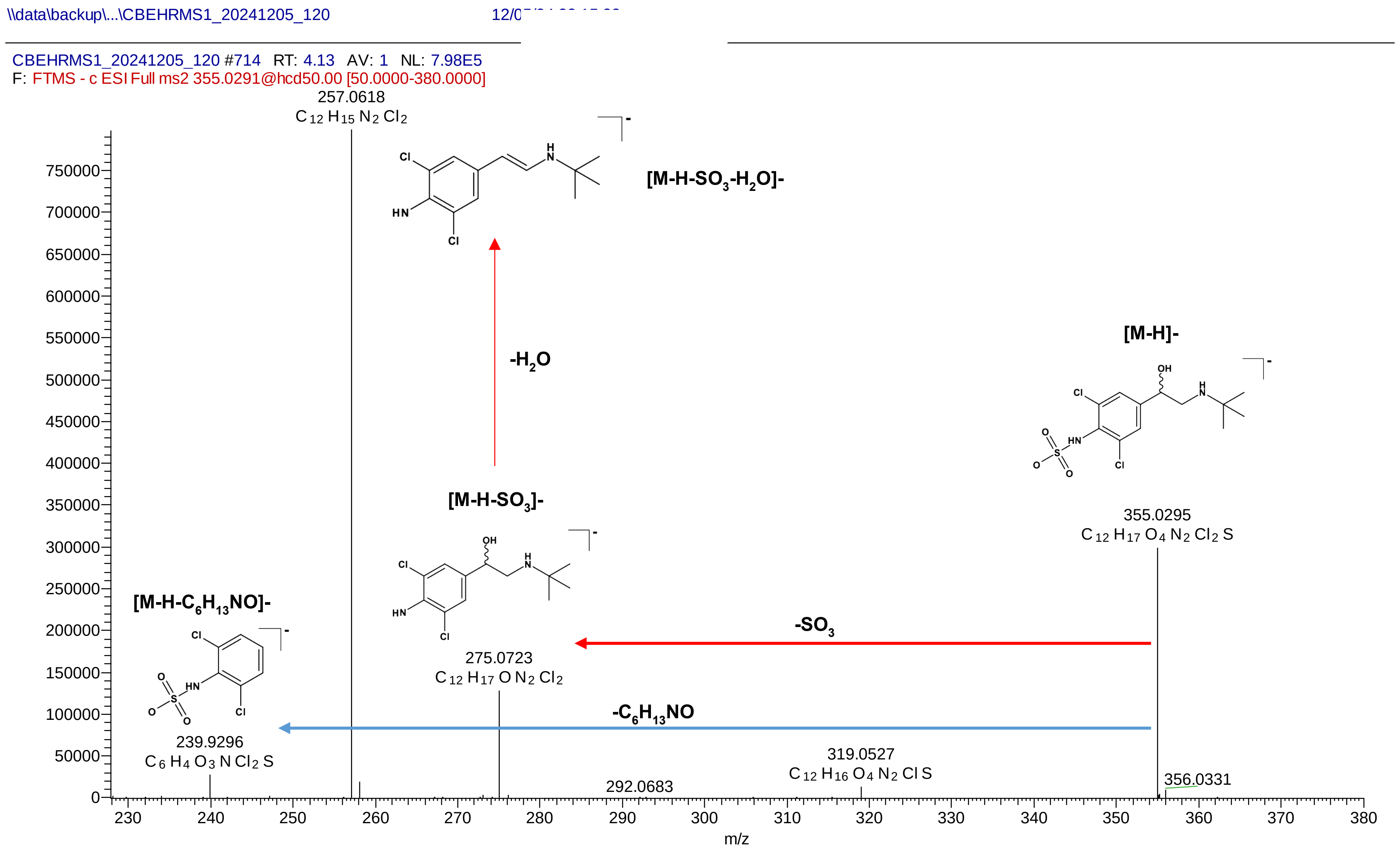

Supplement: Supplementary file 1 — Figure S1: PRM spectra and fragmentation pattern proposed for CLB. ESI+. Figure S2: PRM spectra and fragmentation pattern proposed for N‐OH‐Clb. ESI+. Figure S3: PRM spectra and fragmentation pattern proposed for NO2‐Clb. ESI+. Figure S4: PRM spectra and fragmentation pattern proposed for Gluc1‐2‐Clb. ESI+. Figure S5: PRM spectra and fragmentation pattern proposed for Gluc1‐2‐Clb. ESI−. Figure S6: PRM spectra and fragmentation pattern proposed for Nar‐Met‐Clb. ESI+. Figure S7: PRM spectra and fragmentation pattern proposed for ADBA. ESI−. Figure S8: PRM spectra and fragmentation pattern proposed for ADOA. ESI−. Figure S9: PRM spectra and fragmentation pattern proposed for SO3‐Clb. ESI–. [file DTA-17-2314-s001.pdf]
